# Editorial: Extremophiles in biotechnology: challenges and advancements in sustainable applications

**DOI:** 10.3389/fmicb.2026.1888432

**Published:** 2026-06-19

**Authors:** Ragini Bodade, Rosa María Martínez-Espinosa, Obulisamy Parthiba Karthikeyan, Ameeta Ravikumar, Ajay Kumar

**Affiliations:** 1 Life Science Division, Institute of Advanced Study in Science and Technology, Guwahati, Assam, India; 2 Academy of Scientific and Innovative Research (AcSIR), Ghaziabad, India; 3 Applied Biochemistry Research Group, Multidisciplinary Institute for Environmental Studies “Ramón Margalef”, University of Alicante, Alicante, Spain; 4 Austin Elements Inc., Houston, TX, United States; 5 Institute of Bioinformatics and Biotechnology, Savitribai Phule Pune University, Pune, Maharashtra, India; 6 Amity Institute of Biotechnology, Amity University, Noida, Uttar Pradesh, India

**Keywords:** extremophiles, extremozymes and extremolytes, industrial applications, metabolite production, synthetic biology

Microorganisms from extreme environments have received significant attention worldwide for their unique metabolic adaptation and potential uses in various biotechnological processes. Among these, the members of the Archaea domain display some of the most extreme molecular and physiological adaptations, which makes this group of microorganisms highly interesting for applications in biotechnological processes (Moopantakath et al., 2023; Sepe et al., 2025). Abdul Rehman et al. have traced the origin of extremophiles to the early stages of Earth's evolution and linked them to the last universal common ancestor (LUCA), supporting the hypothesis that thermophiles may represent the earliest forms of life on Earth. Their work further highlighted the diverse survival strategies, including structural and functional adaptations of extremozymes, and intra- and extracellular extremolytes formation, and further emphasized their significance for sustainable industrial production, bioremediation, and climate change mitigation. Comparative genomics and metabolic modeling have additionally revealed how extremophiles optimize energy utilization, genome structure, and metabolic networks to survive under harsh environmental conditions (Abdul Rehman et al.).

Extremophiles also possess sophisticated gene-editing systems that facilitate survival under extreme stress conditions. Yang et al. have discussed the CRISPR-Cas system in bacteria and archaea, including its classification, mechanism of action, and applications in gene editing. They further described TnpB, a protein from radiation-resistant *Deinococcus* species closely related to Cas12f, as a promising next-generation micro-editing tool for future biological and biomedical applications (Yang et al.).

In this context, Mazur-Marzec et al. investigated the biotechnological potential of microorganisms isolated from Baltic Sea harbor samples, which comprise diverse communities of diatoms, dinoflagellates, cyanobacteria, bacteria, fungi, and bacteriophages that remain largely underexplored. Their review highlighted the ability of these organisms to produce lipids for biofuel generation, removal of nutrients such as nitrogen, phosphorus, and carbon (organic and inorganic) from wastewater, generation of hydrogen gas, and synthesis of high-value compounds, including nanoparticles/ nanosilica, pigments, polyunsaturated fatty acids, phycotoxins, antibiotics, and industrial enzymes. The author also emphasized the potential role of Baltic viruses in controlling aquatic pathogens and stimulating the production of valuable cyanobacterial metabolites such as nodularin, anabaenopeptins, aeruginosins, and spumigins (Mazur-Marzec et al.).

Nevertheless, extremophilic fungi offer new prospects for sustainable biotechnology. Sliżewska et al. have investigated molecular mechanisms of salt adaptation of fungal strains isolated from Italian and Polish soils. Their enzymatic profiling demonstrated the ability of these fungi to produce biosurfactant, mycotoxin, and industrially important hydrolytic enzymes, including cellulases, xylanases, and amylases. These enzymes therefore have broad industrial applications, particularly in food processing and biomass conversion, whereas the fungi have been revealed to produce limited amounts of mycotoxins (Sliżewska et al.).

Building on advances involving extremophilic enzymes, Stracke et al. developed an efficient enzymatic approach to synthesize the thermophilic extremolyte cyclic-2,3-diphosphoglycerate (cDPG) from 2-phosphoglycerate (2PG) using enzymes 2-phosphoglycerate kinase (2PGK) and cyclic-2,3-diphosphoglycerate synthetase (cDPGS) from *Methanothermus fervidus* (*Mf* ). Their work demonstrated effective overexpression of these enzymes in *Escherichia coli* (MfcDPGS) and their optimized purification strategies, thus providing a scalable platform for industrial cDPG production with potential applications in healthcare, cosmetics, and pharmaceuticals (Stracke et al.).

In parallel, xylanases from extremophiles have also attracted industrial interest because of their efficiency in lignocellulosic biomass conversion under harsh processing conditions. Li et al. reported two potential xylanase genes, Tc15-Xyn6 and Tc15-Xyn10, based on metagenomic sequencing of sediments from the Qiaoquan hot spring in Tengchong, Yunnan Province. Both enzymes were heterologously expressed in *Escherichia coli*, and exhibited high-temperature tolerance and broad pH stability, with 40%−60% of their initial activity under extreme conditions. Further, the hydrolysis products, including xylobiose, xylotriose, and xylotetraose, considerably promoted the growth of *Lactococcus lactis*, signifying their potential applications as prebiotics and feed additives (Li et al.).

Extremophiles have also been reported to synthesize bio-based compounds such as silver nanoparticles, exopolysaccharides (EPS), and metabolites, which have also received significant attention. Prajapati et al. reported the green synthesis of silver nanoparticles using *Kocuria flava* KKYHNGU1 isolated from rooftop solar panels. The synthesized nanoparticles revealed antibacterial, anti-proliferative, and pro-apoptotic activities against the THP-1 monocytic leukemia cell line, as well as strong reductive abilities for the removal of trimethoprim from wastewater and soil. These findings highlighted the significance of microbial cell factories for the sustainable production of high-value nanomaterials (Prajapati et al.).

Meza et al. performed whole-genome sequencing and functional characterization of *Bacillus licheniformis* Tol1 isolated from the Tolhuaca hot spring in Chile. Under optimized conditions, the strain produced 2.11 g L^−1^ of EPS exhibiting cytotoxic, antioxidant, and emulsifying activities, signifying its applications in food, pharmaceutical, and biomedical industries (Meza et al.).

In another similar study by Gallardo et al. *Exiguobacterium* sp. SH31 has investigated the biosorption of rare earth elements (REEs) such as Ytterbium (Y), Praseodymium (Pr), Neodymium (Nd), Gadolinium (Gd), Terbium (Tb), and Dysprosium (Dy) at a concentration of 0.1 to 1 mM at varied pH. The study demonstrated the potential of microbial-mediated recovery of REEs from e-waste leachates through EPS-mediated surface binding, thereby contributing to sustainable electronic waste revalorization strategies (Gallardo et al.).

Another important biomolecule obtained from extremophiles is bacteriorhodopsin, a light-driven proton pump produced by halophilic microorganisms. Joseph et al. optimized the extraction and characterization of bacteriorhodopsin from *Halostagnicola larsenii* TP6 isolated from the Tuticorin salt pans of India. The pigment demonstrated potential applications in food biotechnology and sustainable biosensor development owing to its unique photoelectric conversion properties (Joseph et al.).

Overall, the extremophilic microorganisms represent an invaluable reservoir of novel biomolecules, enzymes, metabolites, and genetic tools with broad applications in industrial biotechnology, environmental remediation, nanotechnology, healthcare, and sustainable development. Continued exploration of extreme environments, together with advances in genomics, metabolic engineering, and synthetic biology, will further unlock the immense biotechnological potential of these remarkable organisms, as well as gain understanding of the presence of live in other planets as aimed by astrobiology-based disciplines.
